# Having a Parent with Early-Onset Dementia: A Qualitative Study of Young Adult Children

**DOI:** 10.1155/2022/7945773

**Published:** 2022-07-31

**Authors:** Hanne Groennestad, Wenche Malmedal

**Affiliations:** Department of Public Health and Nursing, Norwegian University of Science and Technology (NTNU), Trondheim 7491, Norway

## Abstract

**Background:**

Children of a parent with early-onset dementia (EOD) are confronted with losing a parent to a progressive neurodegenerative illness, usually perceived as an older adult illness, which may have a great impact on their lives at a time that is usually preserved for self-development.

**Objective:**

The objective of this study is to explore the experiences and perceptions of young adult children of a parent with EOD, with specific focus on personal lives and family and social relationships in a Norwegian context.

**Methods:**

Semistructured interviews with 10 young adult children between the ages of 19 and 30 years of a parent with EOD were analysed using a thematic analysis.

**Results:**

The analysis identified six main themes. *“Upon discovering dementia, Keeping the family together, Others do not understand, A sense of relief, A need for support”* and *Apprehension for the future*. The participants expressed an overarching feeling of living parallel lives, summarised by the phrase *“We are not in the same boat.”* Furthermore, the themes demonstrated that the participants experienced difficulties with managing new responsibilities, at the same time, as preserving their own lives. They also shared concerns about the future and often experienced a lack of understanding and support from others. Finally, the need for targeted support throughout the illness was a central theme, whereby many felt more comfortable talking with someone with similar experiences or knowledge of their situation.

**Conclusion:**

The findings in this study strengthen the notion that the children of persons with EOD experience a challenging life situation, underlining the need for a person- and family-oriented approach.

## 1. Introduction

Dementia is an acquired, irreversible illness, characterised by progressive and degenerative decline in mental functioning [1], severely impacting both the affected person and their family. Dementia is not just related to old age, as it can also affect younger persons and their subsequent younger caregivers. An onset of dementia under the age of 65 is referred to as early-onset dementia [2]. Like caregivers of older persons with dementia, the main caregivers of a younger person with dementia are reported to take on high levels of caregiving tasks. Research suggests that caregivers of younger persons with dementia encounter unique challenges and issues in their newfound caregiving role [3, 4]. Most researches have focused on the impact of early-onset dementia (EOD) on the main provider of informal support, most often the spouse [5]. However, it is not only the spouse who is affected, as the children are also faced with a new and uncertain situation.

The gradual decline in mental functioning in a parent can adversely influence the interpersonal relationships between family members and, by extension, family dynamics. Children may become occupied with responsibilities to buffer the loss of a parent at a time in their life that is usually reserved for separation from the family unit and self-development [6]. Furthermore, the idiosyncratic nature of EOD may leave children with few others in similar circumstances outside the family to turn to, thereby creating a potential for distress.

Moreover, even though there is growing recognition that EOD is a significant clinical and social problem [7], most studies do not distinguish between family members or caregivers when exploring how EOD affects the family [8]. This makes it difficult to understand the unique experiences of young adults. This paper explores the experiences and perceptions related to being a young adult child of a parent diagnosed with EOD.

## 2. Background

A systematic review found that the prevalence of all subtypes of EOD ranged from 38 to 420 per 100,000 of the population [9]. Moreover, a recent prevalence study from Norway found that the overall prevalence of dementia in the age group 45–64 years was 143 per 100,000 [10], which is higher than previous reports. Studies of life expectancy after diagnosis are inconclusive, but some have indicated that EOD is associated with higher mortality rates compared to late-onset dementia (LOD) [11].

In addition to known variation based on affected brain functions, persons with EOD may also present more atypical symptoms compared to persons with LOD [12]. They may have a more rapid rate of cognitive decline, although this has mostly been studied in persons with early-onset Alzheimer's disease (AD) [13]. The prevalence of non-memory presentations in persons with early-onset AD has, for instance, been found to be five times higher than with late-onset AD [14]. Other studies further suggest that cases of early-onset AD have more prominent difficulties with language, visuospatial skills, and executive functions [15, 16].

The task of providing care for a person with EOD is associated with both physical and emotional burdens, as shown in a review by Svanberg et al. [17]. The review further reports that EOD caregivers experience higher levels of depression and more psychosocial problems than LOD caregivers. The review further suggests that this may be related to the age or stage of life, such as employment problems and financial difficulties. Qualitative studies looking at the impact on spouses have reported that they struggle with the change in their relationship and feel burdened by their sole role as the economic provider for the family [4].

Most previous studies do not separate the spouses from the children, but rather consider all of the next of kin involved in everyday care of the person with dementia as caregivers, which makes it difficult to find research exclusively on the impact on children [8]. However, some of the existing studies indicate that having a parent with EOD may be a significant stressor. Findings of emotional impact have been identified in qualitative studies and have been associated with feelings of sadness and grief [3]. Family conflicts have also been reported in several qualitative studies, whereby children struggle to cope with their sick parent's behaviour and try their best to avoid confrontations [18] or experiences that their non-affected parent avoids or withdraw from the situation which can leave the children with more caregiving responsibilities and feelings of distress [3, 19]. Some studies also suggest that caretakers of persons with EOD experience feelings of isolation. The studies by Johannessen et al. [4] and Flynn and Mulcahy [1], interviewing spouses of persons with EOD, reported that their social network had shrunk due to their caregiving situation. Similar findings have also been suggested by Barca et al. [3]; where young adult children reportedly experienced difficulties with their social networks.

The families of persons with EOD need social and emotional support [17], but a literature review by Van Vliet et al. [20] found that the lack of designated services for persons with EOD was distressing for the caregivers, as they felt angry and guilty about being forced to accept a service intended for older persons. Studies also suggest that children feel overlooked and are not included in conversations regarding their parent's diagnosis or where to find available support [3, 18]. Some reports have stated that information is crucial to plan for the future [3], while others suggest that children might not want information, especially at the beginning, as they can be frightened by the information that they find [18].

Given that EOD as a form of parental illness has until recently received little attention leaves a need for further exploration. Furthermore, the existing literature often does not distinguish between the spouse and younger or older children when exploring how EOD affects the family [8, 20], making it difficult to understand the experiences and perceptions of young adult children.

## 3. Aim

This study aims to explore the experiences and perceptions related to being a young adult child of a parent diagnosed with EOD with specific focus on the impact on personal lives and family and social relationships in a Norwegian context.

## 4. Methods

### 4.1. Design

The study's design is qualitative with persons in-depth interviews using a semistructured interview guide with open-ended questions to create an exploratory atmosphere during the interview [21]. A test interview was conducted. The interview guide also underwent minor changes during the study to adjust the order of the questions. Prompt questions were also included to fill in or request clarification of what was said during the interviews.

### 4.2. Recruitment and Participants

Two inclusion criteria were established for the study, the participant had to

- Be between 18 and 30 years of age
- Have a parent diagnosed with dementia before or at the age of 65

Two main recruitment sources were used. the Norwegian Health Association and the first author's social network. The first author sent an invitation and a description of the study to the Norwegian Health Association, which was then distributed to their social media pages (e.g., closed groups on Facebook) and to leaders of support groups of young caretakers of parents with EOD. Persons belonging to the first author's social network were also given the invitation to the study and were identified as potential participants through word-of-mouth. In total, 13 candidates responded, two candidates did not meet the inclusion criteria and one was turned down due to their geographical location. After interviewing 10 participants and producing 99 pages of transcribed material, we believed that enough data was collected to adequately illuminate the topic. Thus, the final sample consisted of 10 female candidates, seven recruited through the Norwegian Health Association and three from the first author's social network, all from the south of Norway.

### 4.3. The Interviews

All interviews were audio recorded with the participants' consent and conducted between December 2019 and February 2020. The length of the interviews varied between 45 minutes and 90 minutes and was conducted by the first author. Prior to the interview, the participants were briefed on the purpose of the study and the course of the interview. The interviewer further reaffirmed the participants' consent, in addition to asking whether the participants had any questions before the interview, which is in line with Brinkmann and Kvale [22]. The interviewer considered that the interview could cause emotional upset and encouraged the participants to take breaks if needed. The interviews were conducted in the participants' own homes when possible; otherwise, a neutral venue was chosen.

### 4.4. Analyses

The interviews were transcribed verbatim by the first author. Even though the transcription was a time-consuming process, it also became a valuable experience as it offered the opportunity to capture both tone, volume of voice, and line of flow in the interviews, rather than just spoken words, which is highly beneficial for further analysis according to Malterud [23]. All interviews were transcribed in Norwegian, but quotations used in the final paper were translated into English.

Both authors read the transcribed interviews and the transcriptions were analysed for core concepts and subthemes using a thematic analysis [24]. The analysis also followed an inductive approach, meaning that the themes were strongly linked to the data itself. According to Terry et al. [24], the data were therefore not driven by the researcher's theoretical interest in the area or topic. The interviews were analysed with a focus on each participant's experiences and perceptions within their personal lives and social contexts, as well as their relation to service providers.

At the start of the analysis, all of the transcribed interviews, together with initial notes taken after the interviews by the first author, generated a list of ideas and possible themes based on perceived importance [22]. The next stage of the analysis involved creating initial codes from the raw data. A code identifies a feature of the data that appears interesting [25] and the phrase “smallest meaningful unit” is often used as a designation [26]. During this phase of the analysis, the software tool NVivo [28] was used.

In order not to exclude any data, the data were coded for as many potential themes/patterns as possible whilst still removing data perceived as being irrelevant, as recommended by Terry et al. [24]. During this phase, individual extracts of data were also coded several times. This is in line with Malterud [26] and Brinkmann and Kvale [22], who argued that the analysis should be flexible and dynamic, constantly trying to optimise the coding process.

The next stage of the analysis was to take a step back, gaining an overview and identifying how the different codes could combine to form a potential theme [24]. During this phase, a digital mind map was used to organise the codes (containing extracts of raw data) into theme piles. An example of the coding process is shown in Table 1.

## 5. Results

All ten participants were female, ranging from 19 to 30 years of age; only two of them still lived in the family home when the sick parent was diagnosed.  Table 2 shows the detailed information on the participants.

The parent with EOD ranged from 53 to 61 years; four of them were mothers and six were fathers of the participants.  Table 3 shows detailed information on the parents.

### 5.1. Themes

The analysis resulted in 6 main themes and 20 subthemes (Table 4) which described how their parent's dementia affects the participants' relationships with family and friends, their personal life, and their support needs.

#### 5.1.1. Upon Discovering Dementia

Some of the first or most frequent signs of dementia noticed by the participants were lapses in memory or mood swings in their parent. The signs were mostly insidious and appeared inconsistently; many participants therefore attributed the signs to other causes like stress or depression. Others experienced more rapid or dramatic changes in their parent, as explained by one participant.

*“We were sitting outside eating lunch when all of a sudden, he just took his chair and sat it in between my mother and me and told me “no one comes between us;” he was looking at me like I was some kind of threat (P3).”*

First, the road to diagnosis was a period of questioning the changes observed in the parent. Very few participants tried to speak to their sick parent about the symptoms. Some explained that the parent seemed unaware of any change, or that the parent became defensive whenever they were asked about their abnormal behaviour. Some participants said that the family members were divided in their belief that the symptoms were caused by dementia, or as explained by one participant, refused to believe others' observations and used this as a protective mechanism.

*“I was very angry with anyone who implied that my mother had Alzheimer's and I remember that I could come up with excuses for her behaviour. Even though my sister and father told me that my mother most likely has Alzheimer*'*s disease one year before the diagnosis, I refused to listen; I did not want my mother to be sick (P7).*”

Several participants noted a lack of openness regarding the diagnostic process from both parents and reacted with shock when they were told about the diagnosis. However, accounts of shock and disbelief were also evident among participants who had been more involved in the diagnostic process. Some felt angry, frustrated, or bitter over the fact that someone as young as their parent was suffering from dementia. Others felt a deep sadness; one participant especially spoke of how she right away thought she had to plan for a funeral. For some participants, however, the diagnosis provided a sense of relief.

*“It might not be the right thing to say, but it was a relief. Because I now had an explanation for why she behaves the way she does (P9).”*

#### 5.1.2. Keeping the Family Together

As the changes in the parent became more evident, the parent became increasingly dependent on assistance. Many of the participants felt that they were needed or obligated to care for their sick parent.

*“I feel that this is my responsibility; this is my mother, and I will make sure that she has everything she needs. I feel like I owe it to her in a way, to be there for her, like she has been there for me (P9).”*

The level and type of care the participants provided for their parents differed, depending on factors such as family structure and functioning, living situations, and level of support from healthcare services. However, it was often not easy to find the best way to identify their sick parent's needs.

*“I want to be there for him, but it is not easy. He never wants to do any of the things I suggest, like going for a walk. I just find it really hard to know exactly how I am supposed to be there for him (P6).”*

All participants expressed that their relationship with their parent with dementia had changed. *“Everything has changed, she is not my mother anymore; it is an awful thing to say, but I cannot talk to her in the same way I used to (P8).”*

The altered relationship not only meant losing a special person, but also a role-change, as the participants felt that they had undergone a transformation from being a recipient of care to becoming a caregiver.

Even though most spoke about the role-change in negative terms, some also stated that the parental illness and subsequent role-change had some positive aspects, such as increased maturity and improved personal qualities. The participants not only provided support for the sick parent, but also most found it equally important to support other family members. Some participants raised concerns regarding the health and wellbeing of the other parent, and there seemed to be a sense of duty to provide support in order for their healthy parent to be able to maintain their dual role.

*“When our father told us that he wanted to take some time-off one weekend, we (siblings) right away supported and encouraged him to go and told him “We will take care of her, go and have fun.” When he came back, he felt more energised and could be a much better father to us and husband to my mother (P7).”*

As expressed above, the feeling of cooperating and openly communicating with each other to support the healthy parent, or each other, was significant; as a result, some participants stated that they had become closer to their healthy parent and/or siblings. In contrast, some participants spoke of how tension between their parents, small family units, family members who withdrew from the situation, or lack of support from healthcare services, left them with more sole responsibilities.

The participants described, in various ways, how their increasing concerns and/or needs of their sick parent and/or other family members impacted on their personal life and mental state. Some felt that they never had time or energy for their social life, or stated they had to postpone their plans for higher education or even marriage. It seemed like the overall feeling was that their own lives often clashed with the needs of their sick parent and/or other family members.

*“It is very tiring because you have a constant bad conscience whenever you do something else. I have in some sense gotten used to it by now, but you do have a bad conscience all the time for not doing enough (P6).”*

#### 5.1.3. Others Do Not Understand

When others learned about their parent's diagnosis, many participants had met with reactions of shock and disbelief. It seemed to be a common experience that others felt uncomfortable speaking with participants about their parent's illness, which led some to feel unsupported or not understood. *“I think that they do not understand what it means to have a parent with dementia. But they do not talk to me either, it seems that they do not know how; instead, they just completely avoid the subject (P10).”*

In contrast, a few participants noted that they felt lucky to have persons in their social network who either were knowledgeable of dementia or had similar experiences of parental illness and who could provide them with a kind of emotional and instrumental support that others could not.

That their parent would only become a bit forgetful was a frequent misconception that the participants faced from others. *“Others do not understand how it feels to be a child and see your parent have these episodes of total confusion, or how he can get very angry, childish, and show little sympathy; they do not know that this is happening, they only think you become a bit forgetful (P4).”*

Even though this lack of understanding has resulted in loss of contact with friends and/or family members for some participants, they expressed that they in some way understood them.

*“I think it has been difficult for people because the illness is a bit invisible before you get really ill, it is not like cancer where you can really see that you are sick (P6).”*

#### 5.1.4. Some Sense of Relief

Some of these negative reactions and experiences were reported to cause some participants to feel reluctant talking about their parent's diagnosis. For some, the reluctance to share was also caused by being asked to keep the diagnosis a secret due to the reported feelings of shame by the sick parent. Nevertheless, those participants who chose to be open, or whose parents chose to share their diagnosis, expressed that this provided them with some relief.

*“Since we (mother and participant) agreed that I could tell others about the diagnosis, it has become much easier. If you are having a bad day at work, they will know why; you do not have to explain anything even if you come to work looking like a zombie (P9).”*

Most of the participants and some of the parents with dementia had moved out of the family home, resulting in a completely altered living situation. One participant, where the sick parent had recently moved out the family home, described her experience.

*“I finally feel free (…) and it feels much easier bringing friends home (P4).”*

Many stated that their relationship with the sick parent felt less strained after moving away. *“I think in general that it has been easier for me when I do not live at home. I feel that I have more energy now because I am not in it every day in a sense. When I do come home, I feel less irritated by him (P6).”*

Their sick parent's wellbeing was expressed as a constant cause of concern among most participants. It was important for the participants to feel that their parent had something meaningful to do during the day. However, for a daycare offer to be useful, it was important that it was adapted to the sick parent's needs. When the sick parent had to be in groups of older persons with dementia, they disliked going there, therefore not rendering the same sense of relief.

*“It was difficult for me when we were sending him to a place he did not want to go to. I understand that it is difficult to create an offer that fits everyone, but I do not know, it made me feel bad (P6).”*

#### 5.1.5. A Need of Support

Following the diagnosis, very few participants were offered an opportunity to talk directly to healthcare professionals or were informed of support offers; many felt forgotten or ignored.

As a result, they had to rely on other family members and most often the healthy parent for information, which was described as problematic in several ways. Firstly, the information the healthy parent received regarding dementia, its prognosis, and available support networks was not always passed on to the participants. *“I had to look up things on my own; my father has not been great at asking questions and getting information about what we need to do (P1).”*

“Most participants expressed a need to know as much as possible about the progression of the illness, support offers for themselves and their parents. Many said they searched for this on their own and stated that the Internet was a helpful tool. However, being exposed to detailed and unfiltered information about the illness was described by some as a frightening experience. In addition to a need for information, the participants also expressed a need to meet others in the same situation. *“To have someone my own age to talk to and learn that their experiences are similar to mine would have been nice, because I have always felt that I am the only one who experience this (P2).”*

Those participants that had attended support groups only for young adult children had positive experiences; it is easier to be open with someone in the same situation. However, some expressed a need for one-to-one counselling. “*I wish to have someone who will follow up on you, because you are in a very vulnerable situation when you have a sick parent. I know that my father has Alzheimer's disease and will not be here in the future, but this is a process that takes many years (P10).”*

The timing of the counselling also seemed relevant; for some, a meeting with healthcare professionals immediately after the diagnosis was too early, while others found it useful. Nevertheless, all participants expressed that support offers should become more proactive.

*“You know how to get a psychologist, but it is a hassle to pick up the phone and do it yourself; if you get this “look here is your appointment, all you need is to meet up,” it is so much easier (P7).”*

#### 5.1.6. Apprehension for the Future

The progression of dementia seemed to occupy the participants' thoughts and a common theme was the uncertainty of how fast the progression of the illness would be. This raised a frequent dilemma of how much time they should spend with their parent while minding their own life.

*“I've been worried about not spending last Christmas with my father. At the same time, I do not want to worry whether I should spend Christmas with him every year for the rest of his life. That is how you would think about your 93-year-old grandma; we celebrate this Christmas with her because it might be the last (P2).”*

Some further expressed how this uncertainty caused stress in their everyday life, as some felt that whenever they had the opportunity, they should go and see their sick parent. Some also said it influenced how they planned and said they would think twice before studying abroad for instance. Another difficult aspect of the progression of the illness was the unpredictability of how it would unfold.

*“No one ever told me that he could behave like that (aggressively); I get that, but there is no plan, no “first comes this stage and then the next stage.” I really do not want to think about the future; it just seems like no one can tell me how things will turn out (P4).”*

Not knowing how the illness would progress made many participants feel stressed. Another emotional challenge for the participants was that their parent would forget who they are and this appeared to be something that was too difficult to contemplate.

The participants expressed mixed feelings regarding nursing homes. Many felt concerned about whether the nursing home could facilitate their parent's needs. Therefore, many wanted to have their parent living at home for as long as possible.

*“My father will live at home until he does not understand where he is anymore. It is out of the question putting him in an institution now; he will only be left to rot among 80-year-olds. He also walks several miles per day, so if he only has one hallway to walk in, you will have to put him in a psychiatric ward, because then he really needs help (P3).”*

The thought of their parent in a nursing home was unimaginable for some and evoked feelings of bitterness and frustration, as it felt unfair to visit their parent in an institution many regarded as a place for older persons.

Another concern was related to the burden of caregiving. Some feared that with the progression of the illness, the demands of caregiving would exceed the healthy parent's abilities to cope. As a result, some were fearful that not only their sick parent, but also their healthy parent, would become dependent on them, something that would impact their life for the foreseeable future.

*“I am really worried about the future. I fear that she will not cope, which means that I have to find a balance of living my own life and being there for her; I cannot take mum with me everywhere though-I am supposed to have a life with my boyfriend too (P10).”*

## 6. Discussion

The aim of this study was to explore the experiences and perceptions of young adults with a parent with EOD, with the specific focus on personal lives and family and social relationships.

The participants experienced an overall challenging life situation which led to a need for information and support, something that they sought from other family members, peers, and/or public healthcare services. However, many participants experienced difficulties finding a source of information that was both sufficiently informative and adapted to their needs.

This study also found that the participants' lives were negatively affected by their parents' illness; e.g., by way of interrupting the normal family dynamic and giving the participants different responsibilities compared to their peers. In addition, the role-change impacted their life choices, affecting their transition into adulthood. Furthermore, as the participants' lives differed greatly from that of their peers whose parents were healthy, a recurring and overarching theme was a feeling of living a parallel life. As one participant summarised when comparing their experience with that of their peers. *“We are not in the same boat.”*

### 6.1. Changes in Family Dynamic

The insidious progression of cognitive decline associated with EOD has a profound impact, not only on the affected person, but also on the entire family. As a result of changes in social dynamic and roles within the family, children of parents with EOD pass into adulthood with a parent-child relationship that radically deviates from the expected norm [27].

A central theme in the findings was how the relationship with their parent had suffered due to their diagnosis. According to the participants, the sick parent's personality had changed and they had become more difficult to relate to, which is in accordance with Aslett et al. [29] who suggest that the possibilities of closeness, intimacy, and a meaningful relationship with the parent diagnosed with EOD are strained. Many participants noted that the deterioration of their parent's condition and their gradual removal from their former selves led to a sense of continual loss, with subsequent mixed feelings and emotions. Such a situation, wherein the parent is physically present but psychologically absent, can be described as a psychological ambiguous loss [30] and has been found by previous studies to be a central component in the process of grief experienced by those whose loved ones are affected by dementia [31]. The culmination of this continual and premature sense of loss, as highlighted by many participants, was the feared or actual realisation of no longer being recognized by their own parent. Furthermore, the parent's gradual decline not only led to the aforementioned sense of premature physiological loss, but also led to the loss of a parental figure from which to seek advice or support, on both practical and emotional matters.

This study's findings further suggest that the parent's illness changes the family dynamic, forcing young adults to take more responsibility in keeping the family together, both practically and socially. Some felt a strong sense of filial duty to reciprocate care and support for their sick parent, with mentions of feeling closer to their sick parent, as also found by Aslett et al. [29]. This feeling of being responsible for their parents' emotional wellbeing, also known as emotional parentification, can, in the short term, be an adaptive response to parental illness or a family crisis [32]. However, it is worth noting that emotional parentification in childhood has been found to be a predictor of depression in adulthood [33]. The fact that some participants also muted their own thoughts and feelings, supported by the findings of Gelman and Rhames [27]; shows the importance of research reporting on children's own experiences and not only being represented by their parents. A study of young adult caregivers (18–24 years of age) suggests that it is important to have a perceived choice in caring, especially in a phase where young adults are undergoing extensive changes and taking major decisions on career issues [34]. Moreover, the findings in this study suggest that participants who moved away from their sick parent found that their relationship improved by reducing their daily involvement with the sick parent, as supported by Johannessen et al. [35]. However, moving away from the family home led to feelings of ambivalence, stress, and guilt regarding not being able to contribute as much at home, in accordance with Barca et al. [3].

### 6.2. Transition to Adulthood

The findings of this study suggest that having a parent with EOD can be particularly difficult for young persons' transitioning to adulthood, affecting both short- and long-term life choices. The participants reported that their own life and needs often clashed with those of their sick parent, with some postponing higher education or moving back home as a result. Findings such as these are worth considering as career recognition provides a major foundation for developing a positive view of oneself in young adulthood [36]. It is worth noting that young people in Norway tend to move out from their family home when they are younger than most of their European counterparts [37, 38]. While the majority of young adults in Norway move out by the age of twenty, young adult in southern Europe move out in their late twenties. Young adults in Norway may therefore experience a greater loss of independence when comparing themselves with fellow Norwegian students and friends.

A longitudinal study by Johannessen et al. [35] suggests that resilience gradually evolves as young adults of a parent with EOD master the situation over time. Crucially, this depends on having at least one social relationship with someone who is understanding and supportive of their situation. This is consistent with the findings of the current study, where the importance of having one person in their social network that they could relate to or confide in was emphasised. This is also supported by George et al. [39], who found that social support and increased awareness regarding the psychological burden faced by family members caring for a sick relative, significantly reduced the psychological distress, both for full- and part-time caregivers.

### 6.3. In Need of Targeted Support

The participants in our study felt that their own needs were not specifically considered by the healthcare and/or support services. A dominant experience, also found by Barca et al. [3], was a feeling of being overlooked or ignored by professionals, both in terms of receiving information about their parent's illness and gaining access to available support offers.

In relation to support offers for caregivers of younger persons with dementia, a Norwegian national survey from 2012 revealed few offers within municipality and specialist healthcare services [40]. While the Norwegian Health Association and the Norwegian Centre for Aging and Health, together with other organizations, offer two annual courses in addition to support groups for children of a parent with EOD, there is no formal system ensuring that young adults (over 18 years of age) are contacted by the service system.

To meet someone who could identify and empathise with their own situation was, as previously mentioned, one of the participants' most important needs, something that has also been described in other studies [3, 35]. In line with the findings of Barca et al. [3] and Johannessen et al. [35]; many of the participants valued the social support and guidance from the support groups.

However, in contrast to the findings in Barca et al. [3]; some participants found support groups to be unhelpful. This was particularly in situations where the other participants in the group had parents at a more advanced stage of the illness. Their accounts and experiences did not offer support, but rather contributed to creating more uncertainty in the study participants, rendering them unsure of whether they would be able to cope with their parent's illness in the future. The participants also highlighted the need for support offers throughout the illness and to be included in the diagnostic process; they also stated that support services should be proactive in their approach, which has also been suggested by Millenaar et al. [18].

The need for targeted support can also be extended to the whole family. As described by the participants in this study, meaningful activities for their sick parent provided by the public health services' daycare offer lessened their burden of care. However, specialised daycare offers for persons with EOD are scarce, and typically only available in the most populous municipalities [41]. Most of the offers are designed for older persons with dementia, leaving younger persons with dementia without adequate activities and thus more dependent on their families. The last stage of service from the public healthcare is moving away from the home and into a nursing home. This transition seemed particularly troublesome for many participants. The study by Larsen et al. [42] reports that family caregivers of LOD experienced dilemma regarding the assumed duty of care for the person with dementia and the caregivers' own needs. Feelings such as guilt and a sense of betraying their loved one was prominent, especially when the person with dementia rejected admission to a nursing home. While this was also expressed in this study, the participants seemed particularly troubled with having their parent in a nursing home not suited for younger persons with dementia.

There has been a movement in the government policy towards a whole family approach [43] and such an approach could support parents in retaining their role and promoting positive experiences for the children. This is also important for the healthy parent, as the findings suggested that the non-affected parent often struggles and that the children therefore fear having two dependent parents. Moreover, the participants in this study, similarly to those of the study by Svanberg et al. [44], highlight that healthcare services should strive to cater for the individual needs of young adult children throughout the course of the illness. The fact that some participants experienced a lack of support from their family and friends highlights the need for a public whole family approach to caring. Such an approach has also been supported in the literature to help to maintain cohesiveness in the family [27].

### 6.4. Ethical Approval

The study was assessed and approved by the Norwegian Center for Research Data (NSD) (reference number 529451), ensuring that the anonymity of the participants in the study was sufficiently safeguarded. The study also followed the ethical guidelines of the revised Declaration of Helsinki [45]. In addition, the Regional Committee for Ethics in Medical Research (REC) assessed the study prior to its commencement, concluding that it fell outside their mandate of approval.

### 6.5. Limitations and Strengths of the Study

This study highlights important individual differences in the perception of and response to their circumstances. It provides some insight into how young adults experience and navigate relationships and role changes, what kind of challenges they face associated with EOD during their young adulthood and their need for social and professional support. As the study is qualitative and descriptive in nature, the findings cannot be generalised due to the intrinsic limitations of the study design. However, given the recurring themes across the interviews, it seems plausible that the experiences of the study participants may also be valid for the wider population of young adults with a parent affected by EOD. The study population only consists of female participants; thus, the findings may not necessarily be applicable to males. In addition, some participants had already moved out of their family home prior to their parent's diagnosis, thus perhaps experiencing the expectation of staying at home to provide informal care to a lesser extent, as noted by several study participants.

## 7. Conclusion

### 7.1. Final Conclusion

In summary, the main findings of this study suggest that young adult children with a parent affected by EOD face numerous challenges. Within the family, the parent's diagnosis led to a reversal of roles that deviated from the expected norm and severely altered the family dynamic. In a wider social context, the findings suggest that EOD and its manifestations are poorly understood, which caused an additional burden and led to a perceived lack of emotional support. In combination, these factors, in addition to the timing of the disease, had major implications on important life decisions, affecting the participants lives both in the short- and long-term.

### 7.2. Clinical Implications

The findings suggest that young adult children should be invited to give their opinions and take part in the planning of public services and support offers. Given the isolation experienced by some participants, both from healthcare services and their peers, it is vital for dementia services to become proactive and consider the needs of young adult children. Offers of support should also be available from the start of the diagnostic process throughout the course of the illness.

### 7.3. Suggestion for Future Research

Future research should aim to include male participants, as the existing literature, including this study, has mainly involved female participants. The findings in this study also suggest that longitudinal studies should be conducted in order to look at the long-term consequences of children's experiences and their need for healthcare services over time. Additional research is also needed to understand what adequate and age-appropriate support is required to prevent social isolation and promote emotional and physical wellbeing.

## Figures and Tables

**Table 1:** Example of three final subthemes of the main theme: “keeping the family together.”

Data-extract	Coded for	Subtheme
You have to look out for yourself, because there is such an enormous pressure at home, that if you are pressured at work as well, you will just collapse-P1	Must consider yourself in order to not become overburdened	Considering one's own life

I want to be there for him, but it is not easy. He never wants to do any of the things I suggest, like going for a walk. I just find it really hard to know exactly how I am supposed to be there for him-P6	Finding it difficult to provide social support to sick parent	Assuming responsibility for the sick parent

I mostly just talk with her (healthy parent) about her thoughts and feelings. She has been very upset and cries a lot and has this need to talk. You just try the best you can, but it has not been easy knowing what to say to her, or how I should deal with it-P10	Having to provide emotional support to healthy parent while bottling up own feelings	Having to support the healthy parent

**Table 2:** Characteristics of the participants.

The participants				
Participant	Siblings	Relationship status	Residence	Age
P1	None	Cohabitant	Lives outside family home^*∗*^	30

P2	Three	Cohabitant	Lives outside family home^*∗∗*^	28

P3	One	Single	Lives in family home^*∗*^	22

P4	One	Single	Lives in family home^*∗∗*^	19

P5	Two	Single	Lives outside family home^*∗∗*^	23

P6	Three	In a relationship	Lives outside family home^*∗*^	26

P7	Three	Single	Lives outside family home^*∗*^	24

P8	Seven	Cohabitant	Lives outside family home^*∗*^	29

P9	Four	Cohabitant	Lives outside family home^*∗*^	22

P10	One	In a relationship	Lives outside family home^*∗∗*^	22

^*∗*^Lived outside family home when parent was diagnosed; ^*∗∗*^lived in family home when the parent was diagnosed.

**Table 3:** Characteristics of parent with EOD.

The parent with EOD				
Relationship to participant	Relationship status	Residence	Diagnosis	Age and years since diagnosis
Mother (P1)	Married	Family home	Alzheimer's disease	61 and 3

Father (P2)	Married	Family and nursing home	Frontotemporal dementia	58 and 13

Father (P3)	Married	Family home	Frontotemporal dementia	55 and 3

Father (P4)	Separated	Private apartment	Alzheimer's disease	56 and 4

Father (P5)	Married	Family home	Alzheimer's disease	60 and 4

Father (P6)	Married	Family home	Alzheimer's disease	65 and 4

Mother (P7)	Married	Nursing home	Alzheimer's disease	53 and 5

Mother (P8)	Single	Public apartment	Alzheimer's disease	54 and 2

Mother (P9)	Single	Public apartment	Alzheimer's disease	54 and 2

Father (P10)	Married	Family home	Alzheimer's disease	60 and 4

**Table 4:** Main themes and subthemes.

Main themes	Subthemes
Upon discovering dementia	The first symptoms The road to diagnosis The reaction to diagnosis

Keeping the family together	Assuming responsibility for sick parent Experiencing a role change Having to support the healthy parent Considering one's own life

Others do not understand	It is difficult to relate Forgetting things is the smallest problem You have to see it to believe it

Sense of relief	Choosing to share Moving away Have something meaningful to do

A need for support	Finding information Meeting others in the same situation One to one support

Apprehension for the future	Before it is too late what happens next My parent in a nursing home Two dependent parents

## Data Availability

Data are available on request to the corresponding author, Wenche K. Malmedal, wenche.k.malmedal@ntnu. Note that all data are in Norwegian.
